# Materialism, Self-Objectification, and Capitalization of Sexual Attractiveness Increase Young Chinese Women’s Willingness to Consider Cosmetic Surgery

**DOI:** 10.3389/fpsyg.2018.02002

**Published:** 2018-10-23

**Authors:** Qingqing Sun

**Affiliations:** College of Ideological and Political Education, Henan University of Economic and Law, Zhengzhou, China

**Keywords:** materialism, self-objectification, capitalization of sexual attractiveness, cosmetic surgery consideration, Chinese women

## Abstract

Research reveals significant positive associations between materialism and cosmetic surgery consideration, yet little is known about why this relationship exists. To address this question, the present study examined potential mediators of the links between materialism and cosmetic surgery consideration. A sample of 336 Chinese undergraduate women completed measures of materialism, self-objectification, capitalization of sexual attractiveness, and cosmetic surgery consideration. Correlational analysis showed that materialism, self-objectification, and capitalization of sexual attractiveness were significantly positively correlated with cosmetic surgery consideration. The results of path analyses revealed that higher materialism predicted more willingness to consider cosmetic surgery, both directly and indirectly via higher self-objectification and capitalization of sexual attractiveness.

## Introduction

*Cosmetic surgery* refers to various elective procedures people undergo to change their physical appearance in ways that they perceive to be more desirable (Khoo, 2009). The Chinese demand for cosmetic surgery has increased markedly in recent years. According to International Society of Aesthetic Plastic Surgery statistics, China was ranked third, after the United States and Brazil, in the numbers of surgical procedures and plastic surgeons in 2011 (Hackworth, 2012). A report showed that in 2014, China’s medical beauty market reached $85.2 billion, and the population of Chinese plastic surgery and beauty consumers is becoming more diverse in multiple dimensions, such as age, occupation, income, and geographic area (Analysis and Investment Consulting Report on China Medical Beauty Industry, 2016-2022, 2015). Notably, the proportion of Chinese college students among cosmetic surgery consumers is increasing (Jackson and Chen, 2015a; Wen et al., 2017b). Reports have shown that numerous Chinese college students undergo cosmetic surgery during summer vacation, and the most common cosmetic procedures are double-eyelid surgery and rhinoplasty (Hao, 2017).

However, there are distinct risks from cosmetic surgery, including clinical risks such as infection, hematoma, scars, muscle pain, numbness, and death (Marcus, 2007; Huang et al., 2011), and negative psychological outcomes such as anxiety, depression, and body dysmorphic disorder (Honigman et al., 2004; Huang et al., 2011; Fang et al., 2017). Reports have shown that young Chinese women have insufficient understanding of the risks of cosmetic surgery and lack the psychological ability to cope with these risks (Guo and Xie, 2011), and thus they may be more vulnerable to negative psychological outcomes. Therefore, it is necessary to explore the psychological factors that influence Chinese female college students’ willingness to consider cosmetic surgery.

Many studies have found materialism to predict greater willingness to consider cosmetic surgery. Materialism is an emphasis on the importance of material wealth, including both obtaining and possessing wealth as the key source of happiness and success in life (Richins and Dawson, 1992). In an analysis of consumer culture, Dittmar (2008) stated that consumer culture is characterized by two prominent ideals: body perfection and material success. Body perfection and material success are often linked together in the media, and both emphasize external markers as symbols of an ideal identity. Therefore, materialistic values emphasize looking beautiful as well as having money and expensive property. This suggests that people who endorse materialistic values will adopt or internalize body perfection ideals (Ashikali and Dittmar, 2011; Teng et al., 2016b). In fact, Gu
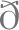
nadóttir and Gar
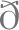
arsdóttir (2014) demonstrated that materialism is positively correlated with the internalization of body perfection ideals for women. And this internalization of beauty standards makes women more likely to consider having cosmetic surgery to increase their attractiveness. Indeed, Henderson-King and Brooks (2009) found that highly materialistic women were more accepting of cosmetic surgery and reported an interest in having more cosmetic surgery procedures.

Since the Chinese economic reform and opening-up policy, with the rapid development of the market economy and deepening of economic globalization, materialistic values have flourished in China, particularly among young people (Wang, 2006). Chinese teenagers are keen on material goods, and luxury consumption is often advocated (Jiang et al., 2016). Furthermore, it is likely that Chinese youth who endorse materialistic values will also want to pursue the perfect body. In fact, studies have found that mass media and advertising often present beautiful faces and ultra-thin bodies (Wang, 2005; Luo, 2012), and young Chinese women who internalize the media ideal of beauty suffer body image disturbances (such as body dissatisfaction) (e.g., Chen and Jackson, 2012) and eating disorders (e.g., Jackson and Chen, 2011). Therefore, it is likely that materialism may also influence young Chinese women’s cosmetic surgery consideration, as those who endorse materialistic values may use cosmetic surgery to reduce the gap between their own appearance and ideal beauty. Therefore, in the present research we sought to investigate the influence of materialism on cosmetic surgery consideration in a sample of Chinese female college students. In addition, given that few studies have further uncovered the internal mechanism behind this association, this research explores two potential mediators of the association between materialism and cosmetic surgery consideration.

The first potential mediator of association between materialism and women’s cosmetic surgery consideration is self-objectification. Self-objectification refers to valuing one’s own body from an external perspective, focusing on observable body attributes (e.g., “How do I look?”) rather than from an internal perspective, focusing on unobservable body attributes (e.g., “How do I feel?”), often manifested by habitually monitoring one’s body and outer appearance (Fredrickson and Roberts, 1997). Although no research has demonstrated that self-objectification mediates the relationship between materialism and cosmetic surgery consideration, previous studies have shown that self-objectification is closely related to both materialism (Teng et al., 2016a,b) and positive attitudes toward cosmetic surgery (e.g., Calogero et al., 2010, 2014). For example, previous literature demonstrated cross-sectionally (Teng et al., 2016b) and experimentally (Teng et al., 2016a) that materialism contributes to the development of self-objectification in a sample of Chinese female college students. It suggests that those who endorse materialistic values are more likely to take an objectifying perspective of themselves.

Moreover, self-objectification is unequivocally reported as a predictor of women’s consideration of cosmetic surgery. For example, Calogero et al. (2014) found that experimentally priming self-objectification increased women’s body shame and willingness to consider cosmetic surgery in the future. In addition, Jackson and Chen (2015a) found that the body surveillance and concern about appearance predicted consideration of cosmetic surgery among young Chinese women and men. Recently, Teng et al. (2016a) reported that degree of body surveillance and appearance management endeavors increased among Chinese college women after they viewed materialistic advertisements. Moreover, increased body surveillance mediated the effect of materialism on women’s appearance management intentions. Therefore, it is plausible that materialism may influence Chinese college women’s cosmetic surgery consideration by affecting their level of self-objectification, a motivator for cosmetic surgery.

The second potential mediator of the association between materialism and cosmetic surgery consideration is the capitalization of sexual attractiveness, which is “the tendency to regard sexual attractiveness as capital for women that can be used as currency to gain rewards such as social and economic successes” (Teng et al., 2016b). Teng et al. (2016b) proposed that in a consumerist society, women may be inclined to regard their sexual attractiveness as capital to gain positive life outcomes. In fact, in modern consumerist society, a woman’s beautiful body or sexual attractiveness is a tangible asset that can manifest identity and social status and allow one to attain more social resources to improve one’s life (Cheng, 2015). For example, slenderness can increase one’s capital, and this capital can be converted into other capital, such as greater social competitiveness, which can improve one’s ability to compete in the marriage market (Tao, 2010). Given that materialism is central to the value systems of a modern consumerist society (Jiang et al., 2016; Teng et al., 2016b), women who endorse materialistic values are more likely to regard their sexual attractiveness as capital to gain benefits. For example, Teng et al. (2016b) found that materialism has positive associations with the capitalization of sexual attraction.

When women regard their sexual attractiveness as capital to gain positive life outcomes, they may be motivated to pursue cosmetic surgery. Generally speaking, a beautiful body will have more value and lead to greater opportunity in the market of symbolic exchange and commodity exchange (Dong, 2016). According to Baudrillard (2000), the female body is sold as a symbol of beauty. As the most beautiful commodity, the female body can be quantified and measured in value, which can be not only produced but also exchanged. A perfect body has a high use value, exchange value, and even symbolic value, which can be directly converted into money or capital and can increase one’s social and economic value (Cheng, 2015). Thus, in order to get a beautiful body, some women choose to have cosmetic surgery, often the most effective and quickest way to move one’s body toward the beauty ideal (Zhang and Wen, 2017). From an economic perspective, cosmetic surgery can be seen as investment in the body; making a profit through investment, realizing the greatest return, is an effort to change one’s life (Cheng, 2015). Indeed, women’s pursuit of a beautiful body actually pursues the “higher value” of attractiveness (Zhang and Wen, 2017). For example, a survey tracking 21 Chinese female cosmetic surgery patients for more than 3 years showed that the improvement of social standing is primary motive for cosmetic surgery. Patients believe that a better body means “more opportunities,” “more confidence,” and “greater life satisfaction” (Zhang and Wen, 2017). In this way, the body becomes an asset for women to use to get opportunities and resources. Therefore, it is plausible that women who have a capitalist viewpoint toward their appearance are more likely to consider having cosmetic surgery, in hopes of improving their appearance to alter their lives. Therefore, we hypothesized that materialism will predict women’s willingness to consider cosmetic surgery via capitalization of sexual attractiveness.

In sum, this study was designed to explore how materialism influences young Chinese women’s consideration of cosmetic surgery. The following four hypotheses were tested: Materialism is positively correlated with self-objectification, capitalization of sexual attractiveness, and consideration of cosmetic surgery (Hypothesis 1); materialism predicts women’s consideration of cosmetic surgery (Hypothesis 2); materialism predicts women’s consideration of cosmetic surgery through self-objectification (Hypothesis 3); and materialism predicts women’s consideration of cosmetic surgery through capitalization of sexual attractiveness (Hypothesis 4).

## Materials and Methods

### Participants and Procedures

The sample consisted of 336 women from a large university in Henan, China. Participants ranged in age from 17 to 21 years (*M* = 18.75, *SD* = 0.81). Body mass index ranged from 15.79 to 27.18 kg/m^2^ (*M* = 20.16, *SD* = 2.16). The majority (96.72%) of participants were of normal weight; 3.27% (*n* = 11) had a body mass index of 25 or higher, the typical cutoff for defining overweight.

Data were collected via a web-based survey hosted by Wenjuan xing (a Chinese survey website). Participants were recruited from various elective psychology courses at the university. Participants completed a questionnaire that comprised the measures of materialism, self-objectification, capitalization of sexual attractiveness, and consideration of cosmetic surgery. This study was approved by the Ethics Committee of Henan University of Economics and Law. All participants submitted online informed consent before filling in the questionnaire. Participants received extra credit for their participation.

### Measurements

#### Materialism

Materialism was measured by the 18-item Material Values Scale (Richins and Dawson, 1992). This scale assesses participants’ endorsement of materialistic values (e.g., “My life would be better if I owned certain things I don’t have”). Participants responded on a 7-point Likert scale ranging from 1 (*totally disagree*) to 7 (*totally agree*). Higher scores indicated greater materialistic orientation. The scale has satisfactory reliability and validity in Chinese samples (e.g., Li and Guo, 2009). In this study, α = 0.78.

#### Self-Objectification via Body Surveillance

Body surveillance was measured by the Body Surveillance subscale of the Objectified Body Consciousness Scale (McKinley and Hyde, 1996). This subscale contains eight items that assess the frequency with which participants monitor their physical appearance (e.g., “During the day, I think about how I look many times”). Participants responded to each item on a 7-point Likert scale ranging from 1 (*strongly disagree*) to 7 (*strongly agree*). The scale has satisfactory reliability and validity in Chinese samples (e.g., Jackson and Chen, 2015a). In this study, α = 0.78.

#### Capitalization of Sexual Attractiveness

Capitalization of sexual attractiveness was measured on a 5-item scale (Teng et al., 2016b) that was modified from the Sex Is Power Scale (Erchull and Liss, 2013). This scale assesses women’s tendency to capitalize on their sexual attractiveness (e.g., “Sexual attractiveness can get women what they want”). Participants responded on a 7-point Likert scale ranging from 1 (*totally disagree*) to 7 (*totally agree*). Higher scores indicated greater tendency to capitalize on women’s sexual attractiveness. Teng et al. (2016b) found that all five items loaded on one component in a sample of young Chinese college women. Alphas higher than 0.80 indicated significant correlations with materialism, self-objectification, body surveillance, and appearance contingency of self-worth in Chinese young women. In this study, α = 0.88.

#### Cosmetic Surgery Consideration

Cosmetic surgery consideration was measured by the Consider subscale of the Acceptance of Cosmetic Surgery Scale (Henderson-King and Henderson-King, 2005). Following previous research (Calogero et al., 2014; Jackson and Chen, 2015a), the 5-item Consider subscale assesses the general intention to pursue cosmetic surgery (e.g., “I have sometimes thought about having cosmetic surgery”). Participants responded on a 7-point Likert scale ranging from 1 (*strongly disagree*) to 7 (*strongly agree*). Higher scores indicated greater intention of having cosmetic surgery. The scale has satisfactory reliability and validity in Chinese samples (e.g., Jackson and Chen, 2015a). In this study, α = 0.90.

#### Demographics

Participants were asked to indicate their age, weight, height, and ethnicity. Body mass index (in kg/m^2^) was calculated on the basis of self-reported height and weight.

#### Data Analysis

To test Hypothesis 1, we used SPSS version 20 to analyze the internal consistency, descriptive statistics, and correlations between the variables.

**FIGURE 1:**
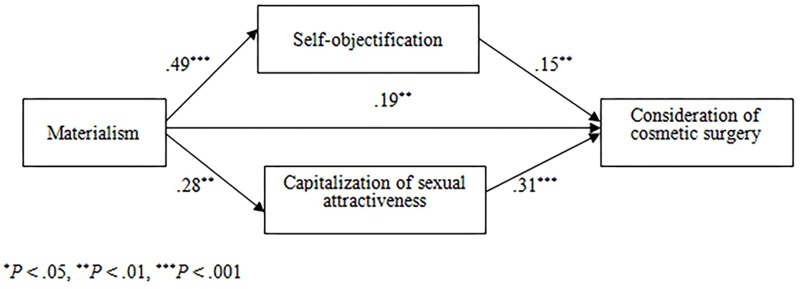
Hypothesized and final model with standardized path coefficients. ^∗^*P* < 0.05, ^∗∗^*P* < 0.01, ^∗∗∗^*P* < 0.001.

To test Hypotheses 2–4, we first used structural equation modeling to test the hypothesized model (Figure 1). Maximum likelihood estimation was carried out in Amos version 17.0. All variables were treated as observable variables. The fit of the model to the data was determined via the four indicators recommended by Hu and Bentler (1999): the comparative fit index (CFI), the Tucker–Lewis index (TLI), the standardized root mean square residual (SRMR), and the root mean square error of approximation (RMSEA). Values of. 95 or higher for the CFI and TLI, 0.08 or lower for the SRMR, and 0.06 or lower for the RMSEA indicate a good fit to the data (Hu and Bentler, 1999). Next, we used the PROCESS SPSS macro (Hayes, 2013) to further test the significance of indirect effects proposed in Hypotheses 3 and 4. Bootstrapping analyses were used with 5,000 bootstrap samples to compute 95% bias-corrected confidence intervals. The indirect effect estimate, and consequently the mediation, is significant if its bias-corrected 95% confidence interval does not contain zero.

## Results

Means, standard deviations, and correlations are presented in Table 1. As hypothesized (Hypothesis 1), materialism, self-objectification, capitalization of sexual attractiveness, and consideration of cosmetic surgery were all positively correlated.

**Table 1:** Descriptive statistics and correlation among study variables (*N* = 336).

Variables	*M*	*SD*	1	2	3	4
(1) Materialism	55.74	7.61	-			
(2) Self-objectification	34.51	7.07	0.49^∗∗^	-		
(3) Capitalization of sexual attractiveness	15.35	6.49	0.28^∗∗^	0.13^∗^	-	
(4) Consideration of cosmetic surgery	16.56	7.27	0.35^∗∗^	0.29^∗∗^	0.32^∗∗^	-

*^∗^*P* < 0.05. ^∗∗^*P* < 0.01*.

The model examined the associations between materialism, self-objectification, capitalization of sexual attractiveness, and consideration of cosmetic surgery (see Figure 1). Results indicated a good fit of the data to the proposed model: χ^2^/*df* = 0.004, CFI = 1.000, TLI = 1.030, SRMR = 0.001, and RMSEA = 0.000. All paths attained significance. Tests of the indirect effects indicated that both self-objectification (indirect effect = 0.075, *SE* = 0.029, 95% CI [0.020, 0.136]) and capitalization of sexual attractiveness (indirect effect = 0.064, *SE* = 0.018, 95% CI [0.033, 0.107]) mediated the materialism and consideration of cosmetic surgery links. Therefore, Hypotheses 2–4 are supported.

## Discussion

The current study investigated the association between materialism and cosmetic surgery consideration in a sample of young Chinese college women. Results generally suggest that women with higher materialist aspirations are more willing to consider cosmetic surgery. Moreover, materialism predicts women’s cosmetic surgery consideration through self-objectification and capitalization of sexual attractiveness.

First, our results corroborate the positive link between materialism and cosmetic surgery consideration (Henderson-King and Brooks, 2009). Materialism predicts greater willingness to consider cosmetic surgery among female college students in China, which supports Hypothesis 2. These findings are consistent with previous research findings that women who endorse materialistic values may adopt body perfection cultural ideals (Ashikali and Dittmar, 2011; Gu
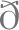
nadóttir and Gar
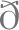
arsdóttir, 2014; Teng et al., 2016b). Such internalization of body perfection values increases women’s dissatisfaction with their bodies (Gu
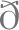
nadóttir and Gar
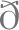
arsdóttir, 2014) and thus makes them more likely to attempt to improve their appearance with cosmetic surgery (Henderson-King and Brooks, 2009).

Consistent with Hypothesis 3, our results also show that self-objectification mediates the relationship between materialism and women’s cosmetic surgery consideration, suggesting that the more materialistic women are, the more likely they are to take an objectifying perspective toward themselves, which in turn leads to a greater intent to pursue cosmetic surgery. This finding is in line with earlier research in which materialism increased Chinese female undergraduates’ self-objectification tendency and appearance management intentions, and the increased body surveillance mediated the effect of materialism on women’s appearance management intentions (Teng et al., 2016a). According to previous studies (Ashikali and Dittmar, 2011; Gu
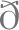
nadóttir and Gar
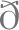
arsdóttir, 2014), women who were high in materialistic values were more likely to internalize and pursue the closely associated cultural standard of “body perfection.” Therefore, they more easily developed an objectifying perspective toward themselves, and they constantly monitored whether their body conformed to the perfect body (Teng et al., 2016a,b). This body monitoring may reveal gaps between women’s appearance and ideal beauty, leading them to accept cosmetic surgery as means of changing their physical appearance (Calogero et al., 2010).

Finally, our results also showed that capitalization of sexual attractiveness mediated the relationship between materialism and cosmetic surgery consideration, which supports Hypothesis 4. This finding suggests that women who endorse materialistic values are more likely to regard their sexual attractiveness as capital to gain positive life outcomes, which in turn increases cosmetic surgery intent. These results extend previous research by including a novel construct, capitalization of sexual attractiveness, to explore how materialism relates to cosmetic surgery consideration among college women. To our knowledge, the only published study to explicitly test the capitalization of sexual attractiveness found a positive correlation between materialism and the capitalization of sexual attraction (Teng et al., 2016b). In a consumerist society, the female body is consumed and capitalized, and the sexual attractiveness of women is a kind of capital that can be transformed into economic, political, and cultural capital (Cheng, 2015). It makes sense that women who subscribe to a capitalist ideology are more likely to take a capitalist viewpoint toward their appearance in exchange for more success. Thus, women may choose to have cosmetic surgery to increase their sexual attractiveness to maximize the benefits. Indeed, a content analysis of the website advertisements of six large cosmetic hospitals in China showed that they often promoted cosmetic surgery by linking sexual attractiveness to a better life (Luo, 2012). They suggest that women can improve their lives by changing their appearance. Furthermore, a survey found that the reason most women choose to have cosmetic surgery is that they believe getting a beautiful body will bring various benefits, such as pleasing others or maintaining marital happiness (Zhang and Wen, 2017). Moreover, a report showed that many Chinese college students resort to cosmetic surgery before graduating from school to bolster their job prospects (He, 2009). Clearly, women are pursuing the increase in economic and social capital that physical changes may bring, not just an improvement in appearance *per se*. Our study is the first to demonstrate the role capitalization of sexual attractiveness plays in influencing women’s cosmetic surgery consideration.

Our results confirm the two mediating mechanisms that explain how young women’s values and beliefs within consumerist cultures may drive their interest in cosmetic surgery. These findings extend previous research by exploring the psychological processes behind willingness to undergo cosmetic surgery (e.g., Sharp et al., 2014; Wen et al., 2017a) and have cross-cultural significance. Moreover, this study has practical implications. First, our results show that materialism is an antecedent of cosmetic surgery consideration and self-objectification, both of which have been demonstrated to cause numerous harmful outcomes in women (e.g., Honigman et al., 2004; Moradi and Huang, 2008). For example, cosmetic surgery often causes psychological complications, such as anxiety (Huang et al., 2011), depression (Honigman et al., 2004), and body dysmorphic disorder (Fang et al., 2017). Self-objectification and the concomitant body surveillance have been demonstrated as risk factors for body image disturbances (Knauss et al., 2008; Jackson et al., 2015), disordered eating (Peat and Muehlenkamp, 2011; Jackson and Chen, 2015b), and depression (Jones and Griffiths, 2015). Thus, some intervention programs to help young women reduce or reject materialistic values may have potentially far-reaching benefits. Second, our findings support calls for changes in how society evaluates women, toward an emphasis on women’s abilities and qualities rather than appearance (Teng et al., 2016b). When society views women in a comprehensive way, there is no need for women to improve their lives by changing their appearance. Future interventions also should be encouraged to improve women’s confidence through their ability to succeed.

The findings of the present study should be interpreted in light of a number of limitations. First, because the sample consisted of Chinese female university students, the results may not be generalizable to women across the age and ethnicity spectrum. It is noteworthy that although women are the main recipients of cosmetic surgery, men’s demand for plastic surgery is also growing (Jackson and Chen, 2015a; Wen et al., 2017b). Thus, future research on a larger and multinational sample is needed to validate this mediational model. Second, our data were collected at a single time point. Thus, no causal claims can be made about the relations between the variables. Future research with longitudinal or experimental designs is warranted. Finally, only the Acceptance of Cosmetic Surgery Scale Consider subscale was used; the Intrapersonal and Social subscales were not used. Of note, the Social subscale assessed social motives to have cosmetic surgery, such as the desire to garner favorable evaluations from others (Henderson-King and Henderson-King, 2005), which may have overlapped and been conflated with the construct of capitalization of sexual attractiveness in this study. However, capitalization of sexual attractiveness emphasizes the general belief that sexual attractiveness can bring about benefits (Teng et al., 2016b), which may affect women in many ways, and cosmetic surgery may be just one of the consequences. For example, Teng et al. (2016b) found that capitalization of sexual attractiveness plays a role in women’s development of self-objectification. The relationship and distinction between these two constructs warrant further investigation.

## Conclusion

This study demonstrates a mediational model indicating that materialism can predict young Chinese women’s cosmetic surgery consideration either directly or indirectly through self-objectification and capitalization of sexual attractiveness. These results extend our understanding of how women’s own values and beliefs within consumerist cultures may drive young adults’ interest in cosmetic surgery.

## Author Contributions

QS carried out the experimental work and the data collection, interpretation, and wrote the manuscript.

## Conflict of Interest Statement

The author declares that the research was conducted in the absence of any commercial or financial relationships that could be construed as a potential conflict of interest.
